# Atypical right diaphragmatic hernia (hernia of Morgagni), spigelian hernia and epigastric hernia in a patient with Williams syndrome: a case report

**DOI:** 10.1186/1752-1947-3-7

**Published:** 2009-01-07

**Authors:** Farhan Rashid, Ramakrishna Chaparala, Javed Ahmed, Syed Y Iftikhar

**Affiliations:** 1 Division of GI Surgery, Clinical Sciences Wing, The Medical School, Derby City General Hospital, Uttoxeter Road, Derby, DE22 3DT, UK; 2 Derby City General Hospital, Uttoxeter Road, Derby, DE22 3NE, UK

## Abstract

**Introduction:**

Williams syndrome is rare genetic disorder resulting in neurodevelopmental problems. Hernias of the foramen of Morgagni are rare diaphragmatic hernias and they mostly present on the right side, in the anterior mediastinum. They are usually asymptomatic and are difficult to diagnose, especially in patients with learning disabilities.

**Case presentation:**

This 49-year-old woman with Williams syndrome, cognitive impairment and aortic stenosis presented to physicians with right-sided chest pain. She had previously undergone repair of her right spigelian and epigastric hernia. Her abdominal examination was unremarkable. Chest X-ray suggested right-sided diaphragmatic hernia and pleural effusion for which she received treatment. The computed tomography scan showed a diaphragmatic hernia with some collapse/consolidation of the adjacent lung. Furthermore, the patient had aortic stenosis and was high risk for anaesthesia (ASA grade 3). She underwent successful laparoscopic repair of her congenital diaphragmatic hernia leading to a quick and uneventful postoperative recovery.

**Conclusion:**

These multiple hernias suggest that patients with Williams syndrome may have some connective tissue disorder which makes them prone to develop hernias especially associated with those parts of the body which may have intracavity pressure variations like the abdomen. Diaphragmatic hernia may be the cause of chest pain in these patients. A computed tomography scan helps in early diagnosis, and laparoscopic repair helps in prevention of further complications, and leads to quick recovery especially in patients with learning disabilities. In the presence of significant comorbidities, a less invasive operative procedure with quick recovery becomes advisable.

## Introduction

Congenital diaphragmatic abnormalities occur in 1/2000 to 1/4000 births [1]. The most common defect is congenital diaphragmatic hernia. Morgagni's hernia is a rare cause of diaphragmatic hernia. It is usually described as an anterior diaphragmatic defect occurring on the right side and located in the anterior mediastinum because of the retrosternal location of the foramen of Morgagni [2]. These hernias are usually diagnosed incidentally when the patient has reached adulthood, or when they become symptomatic due to intestinal involvement (occlusive symptoms) or when they present with respiratory distress [3]. The contents of the hernia usually comprise omental fat and bowel [2].

Williams syndrome is caused by a deletion in the 7q11.23 region which includes at least 17 genes, resulting in a neurodevelopmental disorder [4]. This case report discusses a 49-year-old woman with Williams syndrome and three different types of hernias including an atypical right diaphragmatic hernia incidentally diagnosed on chest – X-ray.

## Case presentation

**Figure 1:**
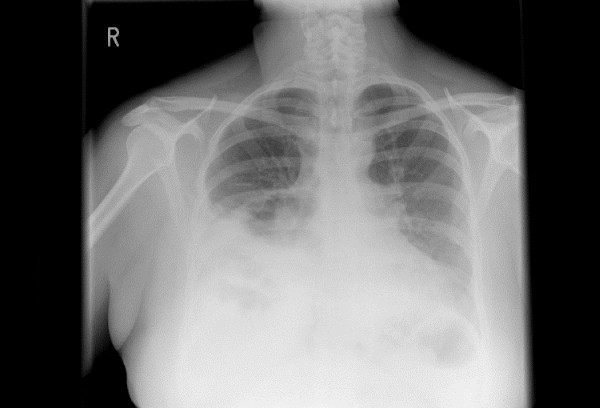
**Chest radiograph (posterior-anterior and lateral view)**. An anterior diaphragmatic hernia containing some elements of the right colon. The heart and mediastinal contours show no gross abnormality. No obvious hilar mass. No lobar collapse or consolidation. No obvious intrapulmonary mass.

**Figure 2:**
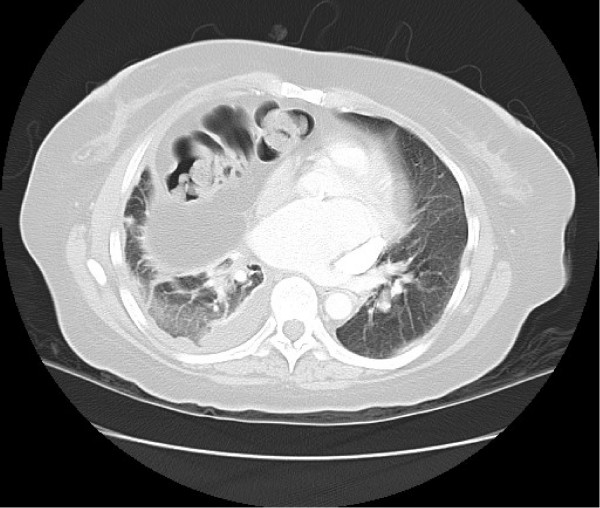
**Axial computed tomography scan of the chest and abdomen demonstrating plate atelectasis in the right upper lobe presumably as a consequence of the longstanding right-sided anterior diaphragmatic hernia of Morgagni which displaces the heart to the left**.

This 49-year-old woman with Williams syndrome, cognitive impairment and aortic stenosis presented to the physicians with right-sided chest pain. In the past, she had undergone repair of her right spigelian and epigastric hernias. Her abdominal examination was unremarkable. Chest – X-ray (Figure 1) suggested right-sided diaphragmatic hernia and pleural effusion for which she received treatment. Pleural aspirate showed neutrophils, lymphocytes and reactive mesothelial cells. There were no malignant cells. A computed tomography (CT) scan (Figure 2) showed a diaphragmatic hernia with some collapse/consolidation of the adjacent lung.

**Figure 3:**
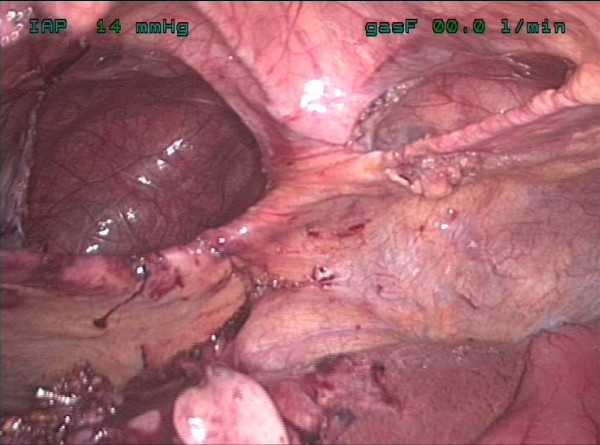
**Intra-operative image of the abdominal cavity showing anterior diaphragmatic hernia above the liver**.

She was considered to be a high-risk patient for general anaesthesia. Her endotracheal intubation was difficult because of her small mouth, receding chin and short neck and she required a bougie to guide the endotracheal tube. In addition to her aortic stenosis, she also had a significantly dilated left atrium, mild mitral stenosis with mild regurgitation, pulmonary hypertension and markedly dilated coronary sinus. The left ventricle was concentrically hypertrophied and the ejection fraction was > 50%. The peak and mean gradients across the aortic valve were 52.7 and 29.5 mmHg, respectively. The electrocardiogram (ECG) showed sinus rhythm with partial right bundle branch block (RBBB) and minor inferior and lateral T wave changes. Despite her significant comorbidities, her persistent symptoms warranted surgical intervention and laparoscopic surgery was preferable to an open repair. So she underwent laparoscopic repair (Figure 3) of her congenital diaphragmatic hernia. Transverse colon and omentum were reduced from the right chest into the abdomen. Intraperitoneal gortex sutures were used to close the defect and the defect was reinforced with surgical porcine mesh and prolene. She made a quick and uneventful postoperative recovery.

## Discussion

Congenital diaphragmatic hernias are a rare form of diaphragmatic hernia and present during adult life. They originate from a defect in the septum transversum due to failure of the closure of the pars sternalis with the seventh costochondral arch. This anatomical defect is also known as Larrey's space and usually lies posterior to the xiphoid process on the right side. Usually this defect is small and asymptomatic until adult life due to plugging by the underlying liver or omentum. Visceral herniation of abdominal content into the thorax occurs when this defect enlarges in size, resulting in respiratory or abdominal symptoms. The hernial sac usually contains omentum, transverse colon and rarely stomach [5]. Increasing weight and chronic cough act as stress factors and influence the symptoms of the Morgagni hernia [6]. Patients can present with either abdominal or chest pain, or respiratory distress [6].

In patients with diaphragmatic hernias, the risks of complications is high, in particular gastric volvulus and colonic obstruction [7]. The hernias may be associated with pleural effusion or any neurodevelopmental disorder as described in this patient. They may be misdiagnosed as localized diaphragmatic eventration, right middle lobe collapse, consolidation, pleuropericardial cyst or mediastinal lipoma and are diagnosed incidentally on chest – X-ray as a homogenous mass in the right cardiophrenic angle [6]. In our patient, the hernial contents were mainly in the cardiosternal space with two obvious defects in the diaphragm.

Plain radiography may not show any abnormality in a patient with intermittent herniation. CT is the modality of choice for diagnosis of Morgagni's hernia and diagnosis may be established by the presence of a fatty mass in a cardiophrenic angle along with omental vessels [6].

Symptomatic cases usually benefit from surgical treatment and prevention of complications. Traditionally, surgical repair has been performed by an open (transabdominal or transthoracic) approach. However, recently these hernias have been successfully repaired using a laparoscopic approach [8]. In the presence of significant comorbidity and learning difficulties, traditional open surgery for these patients carries significant risks. A laparoscopic repair requires experience and expertise. Pre-operative imaging can predict the nature of the hernias and the extent of the diaphragmatic defect. If the hernial defect is small, direct suturing can close it. However, if the defect is large as in our reported case, then a mesh is required [9].

Patients with Williams syndrome have elastin gene deletions which result in altered deposition of elastic fibres in the skin and a subclinical dermal phenotype [10]. It has also been suggested that abnormal vocal cord connective tissue elastin may be the cause of mild vocal cord dysfunction [11]. The participation of the extracellular matrix in the development of inguinal hernias has led to the suggestion that a relationship exists between genetic defects of elastic fibres and collagen synthesis [12].

## Conclusion

Patients with Williams syndrome may have a connective tissue disorder which makes them prone to develop intra-abdominal hernias. Chest pain in these patients may not only be due to aortic stenosis but also to diaphragmatic hernias. Simple investigation such as chest – X-ray may be valuable in detecting these hernias and surgical intervention may relieve the symptoms and prevent complications. In the presence of significant comorbidities, a less invasive operative procedure with quick recovery becomes advisable.

## Consent

Written informed consent was obtained from the next of kin of the patient for the publication of this case report and any accompanying images. A copy of the written consent is available for review by the Editor-in-Chief of this journal.

## Competing interests

The authors declare that they have no competing interests.

## Authors' contributions

FR reviewed the literature and drafted the manuscript. SYI and RC performed the operation. SYI, JA and RC edited the manuscript. All authors contributed intellectual content and have read and approved the final manuscript.
